# PFO closure in ischemic stroke: insights from a single-center real-world cohort

**DOI:** 10.3389/fneur.2026.1792925

**Published:** 2026-05-08

**Authors:** Felix Müller, Arda Civelek, Luis Weitbrecht, Lukas Badura, Kai Helge Schmidt, Florian Schlotter, Philipp Lurz, Stavros V. Konstantinides, Karsten Keller, Klaus Gröschel, Marianne Hahn, Timo Uphaus

**Affiliations:** 1 Department of Neurology, University Medical Center of the Johannes Gutenberg-University, Mainz, Germany; 2 Department of Cardiology, University Medical Center of the Johannes Gutenberg-University, Mainz, Germany; 3 Center for Thrombosis and Hemostasis (CTH), University Medical Center of the Johannes Gutenberg-University, Mainz, Germany; 4 Department of Cardiology, Democritus University of Thrace, Alexandroupolis, Greece; 5 German Center for Cardiovascular Research (DZHK), Partner Site Rhine Main, Mainz, Germany; 6 Medical Clinic VII, Department of Sports Medicine, University Hospital Heidelberg, Heidelberg, Germany

**Keywords:** AF, AS5F, PASCAL, PFO closure, RoPE, stroke

## Abstract

**Background:**

Indications for patent foramen ovale (PFO) closure after ischemic stroke are primarily guided by the Risk of Paradoxical Embolism (RoPE) score and the PFO-associated Stroke Causal Likelihood Classification (PASCAL). Their application in routine care, however, is not well characterized. This study describes real-world management of patients with PFO presenting with ischemic stroke or transient ischemic attack (TIA). TIA cases were included to reflect clinical practice but are interpreted descriptively, as PFO closure is not guideline-supported after TIA alone. As a secondary aim, the “Age, Stroke Severity (NIHSS >5) to Find AF” (AS5F) score was explored for atrial fibrillation (AF) risk assessment.

**Methods:**

This retrospective single-center study included consecutive patients with ischemic stroke or TIA and PFO treated at the University Medical Centre Mainz (2015–2022). TIA was defined clinically by symptom resolution within 24 h due to inconsistent MRI availability. Follow-up was performed via standardized telephone interviews ≥12 months after the index event. Subgroup analyses compared patients by PFO closure status, AF diagnosis, recurrent ischemic events, and age ≥60 years.

**Results:**

Among 188 patients, 62 underwent PFO closure. These patients were younger, had fewer cardiovascular risk factors, and more often exhibited embolic PFO features (PASCAL). The RoPE score was higher in the closure group (6.0 ± 1.7 vs. 4.1 ± 1.5; *p* < 0.001). The AS5F score was lower in closure patients but higher in those with AF (p < 0.001). In patients ≥60 years, higher AS5F scores were associated with AF (cut-off 3.8; *p* = 0.026). Despite greater comorbidity, over half of patients with recurrent events remained classified as cryptogenic.

**Conclusion:**

These findings reflect real-world decision-making in PFO-associated stroke and highlight limitations of score-based approaches. While RoPE and PASCAL remain central, structured AF risk assessment may provide additional value, particularly in older patients. The high rate of cryptogenic recurrence suggests that the role of PFO may be underestimated in clinical practice. Given the moderate sample size, especially in elderly patients, these results should be considered exploratory and hypothesis-generating.

## Introduction

It is estimated that approximately one-quarter of the adult population has a PFO, a remnant of fetal circulatory system that connects the right and left atria (1, 2) commonly detected in healthy individuals. Around 359 million individuals aged 20 years and older live in the European Union (3), suggesting that approximately 89.8 million adults may have a PFO. Every year, roughly 1.1 million people in the European Union suffer from a stroke (4). According to a press release of the German Stroke Foundation in 2023, around 80% of these strokes occur in individuals aged 60 or older and only the minority of 20% are younger than 60 years in Germany, while in the European Union approximately 240.000 cases involve patients under the age of 60 (5). Alongside carotid artery dissection, PFO represents one of the two most frequent etiologies of ischemic stroke in young adults (6).

In 30–40% of the ischemic stroke cases, the underlying cause remains unclear. Many studies have shown a link between cryptogenic stroke and the presence of a PFO in both younger and older patients, although individuals aged 60 or older made up generally less than 1% of the study participants. However, current data indicate that the presence of a PFO alone does not increase the lifetime risk of ischemic stroke (7–9). A meta-analysis by Mojadidi et al., which included the CLOSE, RESPECT, REDUCE, PC, and CLOSURE trials (*n =* 3,440), found that PFO closure significantly reduces the risk of recurrent stroke compared to medical therapy alone (2.0% vs. 4.5%; RR: 0.42; 95% CI: 0.20–0.91; *p* = 0.027) (10).

The “Risk of Paradoxical Embolism (RoPE) score” is used to evaluate the likelihood that a PFO is causally related to a cryptogenic stroke (11). The RoPE score uses patient’s age, conventional risk factors for stroke, and a history of previous stroke, so that the RoPE score decreases with increasing age and burden of vascular risk factors. The RoPE score can be further refined using the “PFO-associated Stroke Causal Likelihood Classification (PASCAL),” which combines the RoPE score with functional and structural criteria of the PFO such as shunt size, the presence of an atrial septal aneurysm, or evidence of venous thromboembolism to optimize risk stratification (12). The PASCAL classification categorizes the probability of stroke due to PFO as definite, probable, highly probable, possible, and unlikely. A thorough diagnostic work-up is therefore essential to avoid attributing strokes to uncertain aetiologies (11, 12, 13–15).

A well-documented complication after PFO closure is the onset of atrial fibrillation (AF), with the majority of cases being transient and resolving spontaneously within the first 12 to 45 days post-procedure (16, 17). Nevertheless, a higher rate of AF occurring beyond the aforementioned periprocedural period was observed compared to those managed conservatively (18). Considering this, there are significant implications in identifying stroke patients who are at an elevated risk of developing AF, irrespective of PFO closure, due to the AF-related risk of recurrent stroke. Evidence suggests that patients with a RoPE score below 7 who do not have any high-risk features per PASCAL classification - such as atrial septal aneurysm or large shunt- are unlikely to benefit from closure and may face increased risk of AF (13). Conversely, patients with both a high RoPE score and high-risk PFO morphology show the greatest absolute risk reduction from closure, while those with intermediate profiles require individualized assessment. This underscores the importance of meticulous risk stratification in guiding treatment decisions.

This single-center cohort study examines the management of patients with ischemic stroke or transient ischemic attack (TIA) associated with PFO in routine clinical practice. Because diffusion-weighted magnetic resonance imaging (MRI) was not consistently available, a time-based, purely clinical definition of TIA—requiring symptom resolution within 24 h—was necessarily applied. The study furthermore evaluates predictors for development of AF in these patients and whether the “Age, Stroke Severity (NIH Stroke Scale > 5) to Find AF”(AS5F) score may be applied as a tool for risk-stratification for AF development in the decision for or against PFO closure. The AS5F score indicates the probability of previously undetected AF being detected in a 72-h long-term ECG following an ischemic stroke or TIA (19). Furthermore, it examines predictors of recurrent strokes in PFO patients.

The current guidelines on the diagnosis and management of PFO after stroke issued by the European Stroke Organisation (ESO) states that reliable data on the effectiveness of PFO closure in patients aged 60 and above are still lacking (20). As such, PFO closure is not currently recommended for this age group. In addition to the issue of insufficient evidence, it should be considered that the likelihood of alternative causes of ischemic stroke, such as atrial fibrillation or macroangiopathic vascular changes, increases with advancing age. This study cohort also examines patients aged ≥60 who underwent PFO closure to determine how they differ from younger patients and whether decision-making criteria can be established for this subgroup, despite their maximum achievable RoPE score being limited to 6.

The overarching aim of this study is to improve the classification of patients with PFO and ischemic stroke and to support the clinical decision-making regarding interventional PFO closure.

## Methods

### Study design and patient population

The Ethics Committee of the State Medical Association of Rhineland-Palatinate approved the conduct of this *study* at the Mainz University Medical Centre under the reference number 2021-15842-1. Of the original 215 patients, 16 withdrew their consent and 11 were lost to follow-up after 90 days during the course of the study (Table 1).

**Table 1:** RoPE score (11) and PASCAL classification system (12).

RoPE Score^a^		PASCAL classification system^b^		
Characteristic	Points	High RoPE Score (≥7)	High-risk PFO (ASA or LS)	PFO-related stroke
No history of hypertension No history of diabetes	1 1	Absent Absent	Absent Present	Unlikely Possible
No history of stroke or TIA Nonsmoker	1 1	Present Present	Absent Present	Possible Probable
Cortical infarct on imaging	1			
Age, y				
18–29	5			
30–39	4			
40–49	3			
50–59	2			
60–69	1			
≥60	0			
Total RoPE score				
Maximum Score	10			
Minimum Score	0			

ASA, Atrial septal aneurysm (defined as ≥10 mm of excursion from midline); LS, Large-Shunt >20 bubbles in the left atrium on transoesophageal echocardiogram; RoPE, Risk of Paradoxical Embolism; PASCAL, PFO-associated Stroke Causal Likelihood Classification; PFO, Patent foramen ovale. ^a^The RoPE Score evaluate the likelihood that a PFO is causally related to a cryptogenic stroke (11). ^b^The PASCAL classification combines RoPE score with functional and structural criteria of the PFO (12).

**Figure 1:**
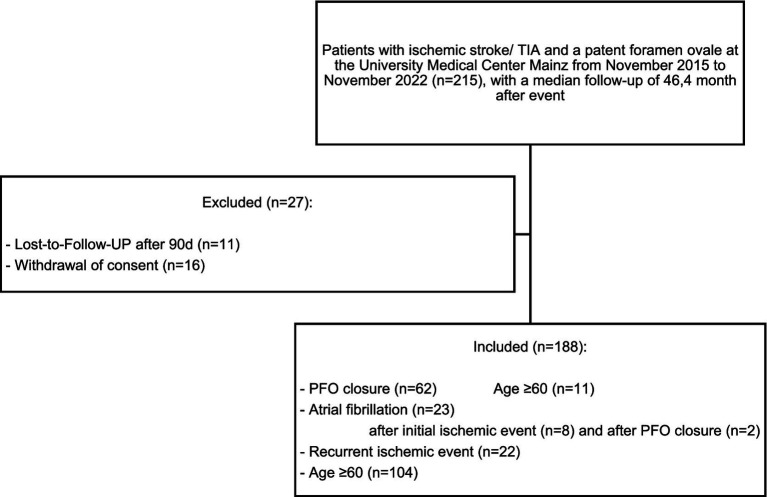
Flow chart of the study group. PFO, Patent foramen ovale; TIA, Transient ischemic attack.

Eligible participants were patients hospitalized with ischemic stroke or TIA and a diagnosis of PFO. Stroke and TIA cases were identified using ICD codes [G45/I63], and PFO was identified using ICD code [Q21.1]. TIA was diagnosed using a time-based clinical criterion, as described in the Introduction. Patients with TIA were included to reflect real-world clinical practice. The diagnosis of PFO was based on transesophageal echocardiography, either performed during the index hospitalization or documented previously in the patients’ medical records prior to admission. The recruitment period spanned from November 2015 to November 2022 (Figure 1).

Data collection concluded in November 2023 with the final follow-up taking place at least 12 months (median 46.4 months) after the initial ischemic event. By this time, 188 patients had provided informed consent. This group included 142 patients identified retrospectively and 46 enrolled prospectively. A descriptive comparison of key baseline parameters between the retrospectively identified (*n =* 142) and prospectively enrolled patients (*n =* 46) suggested that prospectively enrolled patients more frequently underwent PFO closure and had PFO more often attributed as the cause of the index event, likely reflecting improvements in diagnostic workup and interdisciplinary decision-making over the course of the study period. Data collection was carried out by means of a standardized questionnaire.

**Figure 2:**
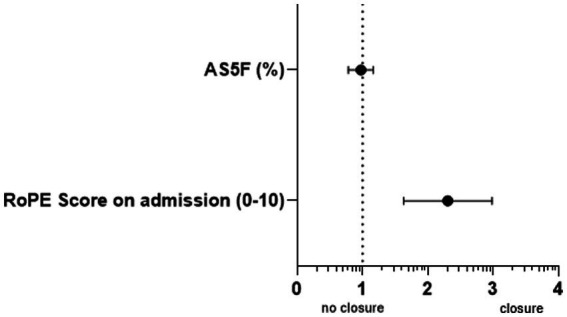
Independent predictors of PFO closure in ischemic stroke. Displayed are adjusted odds ratios with 95% confidence intervals resulting from binary logistic regression. All predictors with *p* < 0.05.

The decision for or against PFO closure was made on an individualized basis within an interdisciplinary setting involving neurology and cardiology, taking into account clinical, morphological, and patient-specific factors (Figure 2).

### Statistics

Nominal variables were presented as absolute frequencies and percentages. Statistical significance was determined using the chi-square test. For continuous and ordinal variables, the normal distribution was tested using the Shapiro–Wilk and Kolmogorov–Smirnov tests. If normal distribution was confirmed, comparisons were made using the t-test and the results were reported as mean values with standard deviation. For non-normally distributed variables, the Mann–Whitney U test was applied, with outcomes reported as medians along with interquartile ranges. The significance level was set at *p* < 0.05.

**Figure 3:**
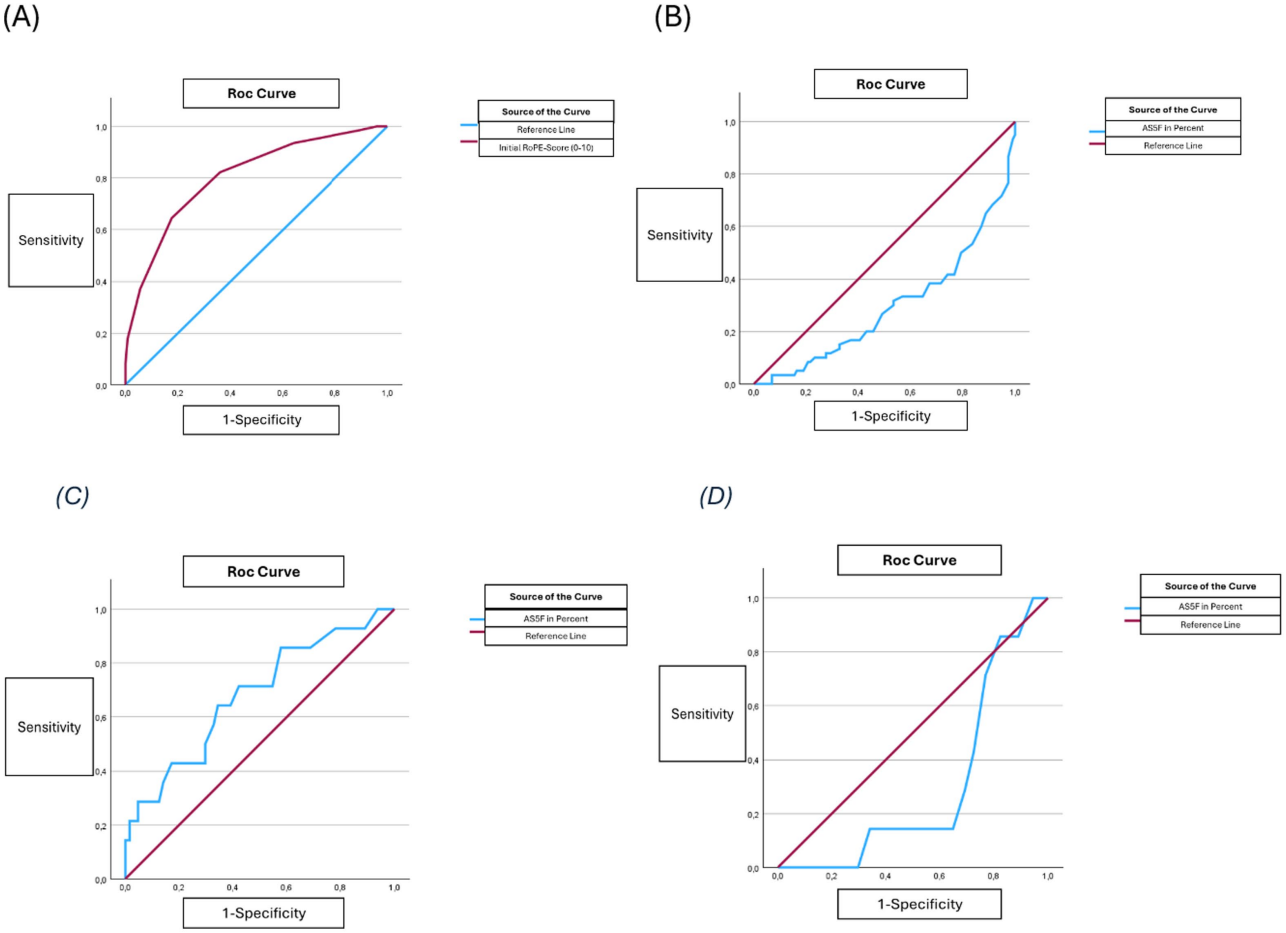
Receiver operating characteristic (ROC) curves illustrating the discriminatory performance of the RoPE score and AS5F score. **(A)** RoPE score predicting PFO closure (AUC = 0.806, *p* < 0.001, 95% CI 0.739–0.873; cut-off = 6). **(B)** AS5F score predicting PFO closure (AUC = 0.706, p < 0.001, 95% CI 0.623–0.789; cut-off = 1.25). **(C)** AS5F score predicting occurrence of atrial fibrillation (AF) in patients aged ≥ 60 years (AUC = 0.680, *p* < 0.026, 95% CI 0.521–0.839; cut-off = 3.8). **(D)** AS5F score predicting AF in patients aged < 60 years (AUC = 0.299, *p* < 0.008, 95% CI 0.151–0.521; cut-off = 0.25).

Binary logistic regression analyses were performed to assess associations between patient and stroke characteristics with PFO closure and with the development of AF. Predictor variables were included in the models if they had shown statistical significance in univariate analyses or were considered clinically relevant. The binary dependent variables were PFO closure (yes/no) and occurrence of AF (yes/no). Receiver operating characteristic (ROC) curves were then generated to evaluate the discriminatory ability of the RoPE score for the decision regarding PFO closure and of the AS5F score for the prediction of AF, with optimal cut-off values for each outcome determined using the Youden index (Figure 3).

All statistical analyses were performed using SPSS (version 27.0 and above).

## Results

*PFO closure*: Overall, 188 patients with ischemic stroke or TIA were included in the present study. Among them, 62 patients (33%) underwent PFO closure, while 126 patients received conservative PFO treatment after the ischemic stroke or TIA. Patients receiving PFO closure were younger at the time of the event (median 51.2 [45.6–59.3] vs. 61 [55–69] years, *p* < 0.001) and showed fewer cardiovascular risk factors and pre-existing conditions such as AF (0% vs. 6.3%, *p* = 0.048). Atrial septal aneurysm (ASA) (21.3% vs. 2.4%, p < 0.001) and other embolic morphologic features such as PFO size (large with > 25 bubbles on echocardiography) (40.0% vs. 19.8%, *p* = 0.001) and dynamics (crossing at rest) (21.1% vs. 16.8%, *p* = 0.018) were significantly more common in patients with PFO closure. The RoPE score (mean of 6 [±1.7] vs. 4.1[±1.5], *p* < 0.001) was significantly higher in the PFO closure group. The AS5F score as a predictive value for AF was significantly lower in the PFO closure group (median of 1.1 [0.5–2.1] vs. 2.0 [1.2–4.05], *p* < 0.001) (for more details see Table 2). The binary logistic regression analysis showed significant independent association of PFO closure with a higher RoPE score (adjusted odds ratio (aOR): 2.625 [CI: 1.866–3.692], *p* = 0.001) and embolic cardiac morphologic criteria such as ASA (aOR: 6.715 [CI: 1.159–38.919], *p* = 0.034). Additional explanatory power of the AS5F score (a predictor for comorbid AF) in PFO closure was tested in a multiple logistic regression model including the RoPE score and the AS5F score, revealing no significant additional association of the AS5F score (aOR: 0.963 [CI: 0.791–1.173], *p* = 0.710) with PFO closure. The discriminatory power as assessed by ROC analysis for the RoPE score as decision criterion for PFO closure was significantly higher compared to the AS5F score (RoPE cut-off: 6 [CI: 0.739–0.873], *p* < 0.001; AS5F cut-off: 1.15 [CI: 0.623–0.789], *p* < 0.001). The difference between ROC curves was statistically significant (*p* = 0.023) (Table 3).

**Table 2:** Single-variable analysis of independent variables in relation to PFO closure.

Variable	Characteristic	PFO closure No (*n =* 126)	PFO closure Yes (*n =* 62)	*p*-value
Demographic data				
Age (years)		66 (59–73)	56 (49–62)	<0.001
Age at the time of the stroke (years)		61 (55–69)	51.2 (45.6–59.3)	<0.001
Risk factors present prior to stroke/TIA				
Hypertension	No (*n =* 91)	52 (52/126, 41.3%)	39 (39/62, 62.9%)	0.005
	Yes (*n =* 97)	74 (74/126, 58.7%)	23 (23/62, 37.1%)	
Smoking	No (*n =* 116)	76 (76/126, 60.3%)	40 (40/62, 64.5%)	0.691
	Yes (*n =* 71)	49 (49/126, 38.9%)	22 (22/62, 35.5%)	
	Unclear (*n =* 1)	1 (1/126, 0.8%)	0 (0/62, 0.0%)	
Diabetes mellitus	No (*n =* 152)	96 (96/126, 76.2%)	56 (56/62, 90.3%)	0.212
	Yes (*n =* 36)	30 (30/126, 23.8%)	6 (6/62, 9.7%)	
Hyperlipidemia	No (*n =* 120)	85 (85/126, 67.5%)	54 (54/62, 87.1%)	0.151
	Yes (*n =* 48)	40 (40/126, 31.7%)	8 (8/62, 12.9%)	
	Unclear (*n =* 1)	1 (1/126, 0.8%)	0 (0/62, 0.0%)	
Comorbidities Present prior to stroke/TIA				
Atrial fibrillation (AF)	No(*n =* 179)	118 (118/126, 93.7%)	61 (61/62, 98.4%)	0.048
	Yes (*n =* 8)	8 (8/126, 6.3%)	0 (0/62, 0.0%)	
	Unclear (*n =* 1)	0 (0/126, 0.0%)	1 (1/62, 1.6%)	
Previous ischemic stroke	No (*n =* 158)	101 (101/126, 80.2%)	57 (57/62, 91.9%)	0.018
	Yes (*n =* 27)	24 (24/126, 19.0%)	3 (3/62, 4.8%)	
	Unclear (*n =* 3)	1 (1/126, 0.8%)	2 (2/62, 3.2%)	
Migraine without aura	No (*n =* 179)	119 (119/126, 94.4%)	60 (60/62, 88.7%)	0.689
	Yes (*n =* 8)	6 (6/126, 4.8%)	2 (2/62, 3.2%)	
	Unclear (*n =* 1)	1 (1/126, 0.8%)	0 (0/62, 0.0%)	
Migraine with aura	No (*n =* 177)	122 (122/126, 96.8%)	55 (55/62, 88.7%)	0.026
	Yes (*n =* 11)	4 (4/126, 3.2%)	7 (7/62, 11.3%)	
	Unclear (*n =* 0)			
Anatomical and etiological features of stroke/TIA				
RoPE score on admission (0–10)		4.1 ± 1.5	6 ± 1.7	<0,001
Cortical involvement of infarction	No (*n =* 109)	79 (79/108, 73.1%)	30 (30/53, 56.6%)	0.035
	Yes (*n =* 52)	29 (29/108, 26.9%)	23 (23/53, 43.4%)	
PFO is the cause of ischemic stroke / TIA	No (*n =* 37)	37 (37/125, 29.6%)	0 (0/62, 0.0%)	<0.001
	Yes (*n =* 41)	10 (10/125, 8.0%)	31 (31/62, 50.0%)	
	Unclear (*n =* 109)	78 (78/125, 62.4%)	31 (31/62, 50.0%)	
Diagnostic workup before PFO closure				
Resting ECG AF	No (*n =* 172)	111 (111/119, 93.3%)	61 (61/61, 100.0%)	0.038
	Yes (*n =* 8)	8 (8/119, 6.7%)	0 (0/61, 0.0%)	
Atrial septum aneurysm (ASA)	No (*n =* 161)	118 (118/125, 94.4%)	43 (43/61, 70.5%)	<0.001
	Yes (*n =* 16)	3 (3/125, 2.4%)	13 (13/61, 21.3%)	
	Unclear (*n =* 9)	4 (4/125, 3.2%)	5 (5/61, 8.2%)	
Continuous long-term ECG AF	No (*n =* 167)	106 (106/117, 90.6%)	61 (61/61, 100.0%)	0.047
	Yes (*n =* 8)	7 (7/117, 6.0%)	0 (0/61, 0.0%)	
	Unclear (*n =* 4)	4 (4/117, 3.4%)	0 (0/61, 0.0%)	
Intracranial Stenosis > 50%	No (*n =* 166)	107 (107/126, 84.9%)	59 (59/62, 95.2%)	0.044
	Yes (*n =* 17)	16 (16/126, 12.7%)	1 (1/62, 1.6%)	
	Unclear (*n =* 5)	3 (3/126, 2.4%)	2 (2/62, 3.2%)	
Character of the PFO				
PFO size	Unclear (*n =* 96)	64 (64/126, 50.8%)	32 (32/60, 53.3%)	0.001
	Small (*n =* 29)^a^	27 (27/126, 21.4%)	2 (2/60, 3.3%)	
	Medium (*n =* 12)^b^	10 (10/126, 7.9%)	2 (2/60, 3.3%)	
	Large (*n =* 49)^c^	25 (25/126, 19.8%)	24 (24/60, 40.0%)	
PFO dynamics	At rest (*n =* 31)	19 (19/113, 16.8%)	12 (12/57, 21.1%)	0.018
	Valsalva (*n =* 73)	57 (57/113, 50.4%)	16 (16/57, 28.1%)	
	Both (*n =* 66)	37 (37/113, 32.7%)	29 (29/57, 50.9%)	
Follow-up after ≥12 months				
AF after stroke/ TIA	No (*n =* 179)	117 (117/125, 93.6%)	62 (62/62, 100.0%)	0.042
	Yes (*n =* 8)	40 (40/126, 31.7%)	0 (0/62, 0.0%)	
Recurrent ischemic event (stroke/TIA)	No (*n =* 165)	106 (106/125, 84.8%)	59 (59/62, 95.2%)	0.038
	Yes (*n =* 22)	19 (19/126, 15.2%)	3 (3/62, 4.8%)	
Age ≥60 at event	No (*n =* 105)	54 (54/126, 42.9%)	51 (51/62, 82.3%)	<0.001
	Yes (*n =* 83)	72 (72/126, 57.1%)	11 (11/62, 17.7%)	
AS5Fscore in %		2.0 (1.2–4.05)	1,050 (0.5–2.1)	<0.001

Nominal variables in percent. Metric and ordinal variables with normal distribution with mean and standard deviation. If no normal distribution, median and 25th and 75th quartile. Significance level = 0.05. AF, atrial fibrillation; ASA, Atrial Septal Aneurysm; AS5F, Age, Stroke Severity (NIH Stroke Scale >5) to Find AF; ECG, Electrocardiogram; PFO, patent foramen ovale; RoPE, Risk of Paradoxical Embolism; TIA, transient ischemic attack. ^a^1–5 bubbles, ^b^6–25 bubbles, ^c^25 bubbles on echocardiography.

**Table 3:** Binary logistic regression of independent variables in relation to PFO closure.

Variable	Adjusted odds ratio (aOR)	95% CI	*p*-value
RoPE score on admission (0–10)	2.625	1.866–3.692	0.001
ASA Unclear			0.039
ASA Yes	6.715	1.159–38.919	0.034
ASA No	4.910	0.719–33.547	0.105
Size of the PFO Unclear			0.057
Size of the PFO Small^a^	0.085	0.012–0.609	0.014
Size of the PFO Medium^b^	0.871	0.102–7.456	0.899
Size of the PFO Large^c^	1.500	0.555–4.053	0.424
Dynamics of the PFO at rest			0.211
Dynamics of the PFO under Valsalva	0.969	0.289–3.248	0.960
Dynamics of the PFO Both	2.178	0.698–6.797	0.180

R^2^ (Nagelkerke) = 0.539. Significance level = 0.05. Univariate variables are significant. CI = confidence interval. A binary logistic regression using forward likelihood shows that the RoPE score alone without the variables included in the RoPE score is significant at *p* < 0.001. ASA, Atrial Septal Aneurysm; PFO, patent foramen ovale; RoPE, Risk of Paradoxical Embolism. ^a^1–5 bubbles, ^b^6–25 bubbles, ^c^25 bubbles on echocardiography.

**Figure 4:**
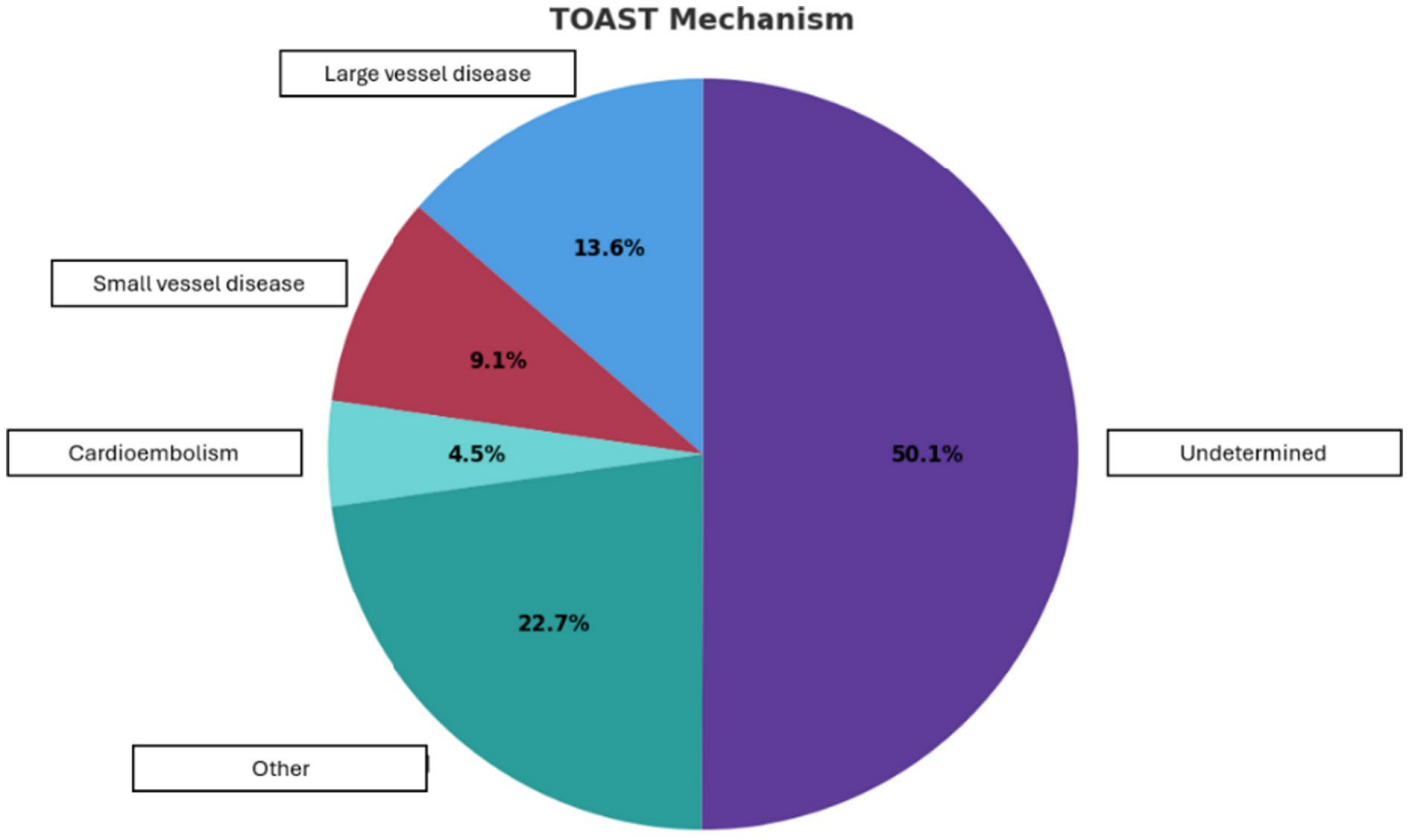
Descriptive presentation of the cause of recurrent ischemic events (*n =* 22) according to the TOAST classification.

*Atrial fibrillation*: In the subgroup of patients with AF (*n =* 23), age was significantly higher than in the patient group without AF (median 66.4 [54.5–74.6] vs. 58.1 [50.7–65.2] years, *p* = 0.006). AF occurred more frequently in male patients (16.7%) compared to female patients (4.6%). The patient population with AF was more likely to have a history of heart failure (8.7% vs. 0.6%, *p* = 0.015) and had an increased mortality during the study period (17.4% vs. 3.7%, *p* = 0.022) compared to patients without AF. The RoPE score was significantly lower (median 3 [2–5] vs. 5 [4–6], *p* = 0.002) and the AS5F score higher (mean of 4.8 [± 5.5] vs. 2.3 [± 2.6], *p* = 0.001) in patients with AF. In binary logistic regression analysis, the RoPE score (aOR: 0.652 [CI: 0.463–0.918], *p* = 0.014) and AS5F score (aOR: 1.125 [CI: 0.985–1.284], *p* = 0.082) were significant independent predictors of the development of AF. Adding enlarged left atrium to the model showed a non-significant association with AF (aOR: 3.275 [95% CI: 0.755–14.212], *p* = 0.113). Also in patients ≥60 years, patients with AF (*n =* 14) had a higher AS5F score vs. no AF (*n =* 64) (mean AS5F of 6.8 [± 5.8] vs. 3.8 [± 2.7], *p* < 0.001). The ROC analysis showed a significant discriminatory power of the AS5F score for AF in patients aged > 60 years (cut-off: 3.8 [CI: 0.521–0.839], *p* = 0.026) (Figure 4).

*Recurrent ischemic event*: Patients who experienced a recurrent ischemic event during follow-up (*n =* 22, 12%) were older at the time of the qualifying event (mean 59.9 ± 16.4 vs. 58.3 ± 11.8 years, *p* = 0.046) and had a higher overall burden of cardiovascular risk factors (Table 4).

**Table 4:** Single-variable analysis of independent variables in relation to the probability of the occurrence of AF.

Variable	Characteristic	AF No (*n =* 165)	AF Yes (*n =* 23)	*p*-value
Demographic data				
Age (years)		62.0 (54.0–69.5)	70.0 (60.0–78.0)	0.015
Age at the time of the stroke (years)		58.1 (50.7–65.2)	66.4 (54.5–74.6)	0.006
Height (cm)		173.6 ± 9.4	178.9 ± 8.5	0.016
Gender	Male (*n =* 120)	100 (100/165, 60.6%)	20 (20/23, 87.8%)	0.016
	Female (*n =* 68)	65 (65/165, 39.4%)	3 (3/23, 12.2%)	
	Diverse (*n =* 0)			
Comorbidities present prior to stroke/TIA				
Heart failure	No (*n =* 185)	164 (164/165, 99.4%)	21 (21/23, 91.3%)	0.015
	Yes (*n =* 3)	1 (1/165, 0.6%)	2 (2/23, 8.7%)	
	Unclear (*n =* 0)			
Anatomical and etiological features of stroke/TIA				
mRS (0–6)		1 (1.0–3.0)	4 (2.0–5.0)	0.049
RoPE score on admission (0–10)		5 (4.0–6.0)	3 (2.0–5.0)	0.002
Diagnostic workup before PFO closure				
Enlarged left atrium	No (*n =* 156)	141 (141/149, 94.6%)	15 (15/21, 71.4%)	0.001
	Yes (*n =* 14)	8 (8/149, 5.4%)	6 (6/21, 28.6%)	
Reduced left ventricular ejection fraction	No (*n =* 172)	153 (153/155, 98.7%)	19 (19/22, 86.4%)	0.005
	Yes (*n =* 5)	2 (2/155, 1.3%)	3 (3/22, 13.6%)	
PFO closure yes or no	No (*n =* 126)	105 (105/165, 63.6%)	21 (21/23, 91.3%)	0.027
	Yes (*n =* 62)	60 (60/165, 36.4%)	2 (2/23, 8.7%)	
Diagnostic workup after PFO closure				
Echocardiography 1 week after PFO closure	No (*n =* 8)	8 (8/57, 14.0%)	0 (0/2, 0.0%)	0.034
	Yes (*n =* 51)	49 (49/57, 86.0%)	2 (2/2, 100.0%)	
Residual PFO shunt	No (*n =* 46)	45 (45/48, 93.8%)	1 (1/2, 50.0%)	0.025
	Yes (*n =* 0)			
	Unclear (*n =* 4)	3 (3/48, 6.3%)	1 (1/2, 50.0%)	
Follow-up after ≥12 months				
Patient deceased	No (*n =* 177)	158 (158/164, 96.3%)	19 (19/23, 82.6%)	0.022
	Yes (*n =* 10)	6 (6/164, 3.7%)	4 (4/23, 17.4%)	
AS5F score in %		2.3 ± 2.6	4.8 ± 5.5	<0.001

Nominal variables in percent. Metric and ordinal variables with normal distribution with mean and standard deviation. If no normal distribution, median and 25th and 75th quartile. Significance level = 0.05. AF, Atrial Fibrillation, AS5F: Age, Stroke Severity (NIH Stroke Scale >5) to Find AF, mRS, modified Rankin Scale; PFO, patent foramen ovale; RoPE, Risk of Paradoxical Embolism; TIA, transient ischemic attack.

PFO closure was significantly less frequent among patients who experienced a recurrent ischemic event (3 [13.6%] vs. 19 [86.4%], *p* = 0.038). Evaluation using the Trial of Org 10,172 in Acute Stroke Treatment (TOAST) criteria showed that most recurrent events were of undetermined etiology (*n =* 11), though four of these could be classified as Embolic Stroke of Undetermined Source (ESUS) and potentially related to PFO, while only one event was attributed to a clearly cardioembolic source.

## Discussion

This single-center cohort study demonstrates that real-world management of patients with PFO and ischemic stroke or TIA largely aligns with current guideline recommendations (20). Both the RoPE score and morphologic features according to the PASCAL classification were significant independent predictors for PFO closure, underscoring the clinical utility of these scoring systems as decision-support tools in determining the indication for PFO closure and estimating the likelihood of a causal association between PFO and cryptogenic stroke (11–13, 21). It should, however, be noted that despite statistically significant group differences, the considerable overlap in score distributions between groups reflects the inherent limitation of score-based decision tools at the individual patient level. While ROC analysis confirms the utility of the RoPE score as a population-level stratification tool, its discriminatory capacity at the level of the individual patient remains limited, which should be considered when translating group-level findings into clinical decision-making.

AF represents a relevant peri-procedural complication following PFO closure (13, 22) and increases the risk of recurrent stroke. A key finding of this study is the potential utility of the AS5F score as a complementary decision criterion for PFO closure, particularly in identifying patients at higher risk of AF who may derive limited benefit from the procedure. While the RoPE and PASCAL scores assess the likelihood that PFO caused the index stroke, they do not directly address the competing risk of AF-related recurrence. The AS5F score fills this gap by estimating the probability of detecting previously undetected AF, a critical consideration given that AF represents both a peri-procedural complication following PFO closure and an independent stroke risk factor.

The biological plausibility of using AF risk as a decision criterion for PFO closure is well-founded. AF-related stroke mechanisms differ fundamentally from paradoxical embolism through PFO: the former results from atrial stasis and thrombus formation, while the latter requires venous thromboembolism and right-to-left shunting. Patients at high AF risk are more likely to experience cardioembolic strokes originating from the left atrium rather than paradoxical emboli, making PFO closure mechanistically less relevant. Additionally, the increased AF incidence following PFO closure—particularly with certain device types—suggests that patients with pre-existing AF susceptibility may experience net harm from the procedure (22, 23).

Integrating the AS5F score into clinical practice would entail minimal additional effort, as it relies on routinely available variables such as age and NIHSS score. Within a stepwise decision framework, patients with cryptogenic stroke or TIA and concomitant PFO would first be evaluated using established tools, namely the RoPE and PASCAL scores. In cases indicating a high likelihood of PFO-related stroke (e.g., RoPE ≥7 and favorable PASCAL classification), the AS5F score could serve as an adjunctive filter. Low AS5F values (<3.8 in patients aged ≥60 years, with potentially different thresholds in younger patients) would favor proceeding with PFO closure, whereas higher scores would argue for prolonged cardiac rhythm monitoring, such as implantable loop recorders, prior to definitive treatment decisions. This approach aligns stroke prevention strategies with the most probable underlying mechanism, whether paradoxical embolism or occult AF.

In the present cohort, patients undergoing PFO closure exhibited markedly lower AS5F scores than those later diagnosed with AF, indicating a lower estimated risk of AF as the primary stroke etiology. This inverse association suggests that incorporating the AS5F score into pre-procedural evaluation may refine patient selection by identifying individuals in whom PFO is more likely to represent the causal mechanism. In particular, a low AS5F score in combination with a high RoPE score and favorable PASCAL features strengthens the justification for closure, whereas elevated AS5F values may signal the need to prioritize extended cardiac monitoring over immediate intervention (Table 5).

**Table 5:** Single-variable analysis of independent variables in relation to the occurrence of re-infarction after closure of a PFO.

Variable	Characteristic	Recurrent ischemic event No (*n =* 165)	Recurrent ischemic event Yes (*n =* 22)	*p*-value
Demographic data				
Age at the time of the stroke (years)		58.3 ± 11.8	59.9 ± 16.4	0.046
Comorbidities Present Prior to Stroke/ TIA				
Earlier ischemic stroke	No (*n =* 157)	144 (144/165, 87.3%)	13 (13/22, 59.1%)	0.003
	Yes (*n =* 27)	19 (19/165, 11.5%)	8 (8/22, 36.4%)	
	Unclear (*n =* 3)	2 (2/165, 1.2%)	1 (1/22, 4.5%)	
Medication before the diagnosis Stroke/TIA				
Statins	No (*n =* 145)	132 (132/165, 80.0%)	13 (13/22, 59.1%)	0.027
	Yes (*n =* 42)	33 (33/165, 20.0%)	9 (9/22, 40.9%)	
Laboratory values on admission				
HbA1c - value (%) on admission		5.5 (5.3–5.8)	5.9 (5.6–6.6)	0.012
Anatomical and Etiological Features of Stroke/ TIA				
Cortical involvement of infractiom	No (*n =* 109)	90 (90/139, 64.7%)	19 (19/21, 90.5%)	0.018
	Yes (*n =* 51)	49 (49/139, 35.3%)	2 (2/21, 9.5%)	
Diagnostic workup before PFO closure				
Echocardiography before PFO closure	No (*n =* 1)	0 (0/164, 0.0%)	1 (1/22, 4.5%)	0.006
	Yes (*n =* 185)	164 (164/164, 100.0%)	21 (21/22, 95.5%)	
Enlarged left atrium	No (*n =* 155)	139 (139/148, 93.9%)	16 (16/21, 76.2%)	0.006
	Yes (*n =* 14)	9 (9/148, 6.1%)	5 (5/21, 23.8%)	
Continuous long-term ECG	No (*n =* 9)	6 (6/164, 3.7%)	3 (3/22, 13.6%)	0.041
	Yes (*n =* 177)	158 (158/164, 96.3%)	19 (19/22, 86.4%)	
Stenosis > 50% of the carotid/ vertebral artery	No (*n =* 146)	134 (134/163, 82.2%)	12 (12/22, 54.5%)	0.006
	Yes (*n =* 37)	27 (27/163, 16.5%)	10 (10/22, 45.5%)	
	Unclear (*n =* 2)	2 (2/163, 1.2%)	0 (0/22, 0.0%)	
Imaging of the intracranial vessels	No (*n =* 1)	0 (0/165, 0.0%)	1 (1/22, 4.5%)	0.006
	Yes (*n =* 186)	165 (165/165, 100.0%)	21 (21/22, 95.5%)	
Intracranial Stenosis > 50%	No (*n =* 165)	149 (149/165, 90.3%)	16 (16/22, 72.7%)	0.005
	Yes (*n =* 17)	11 (11/165, 6.7%)	6 (6/22, 27.3%)	
	Unclear (*n =* 5)	5 (5/165, 3.0%)	0 (0/22, 0.0%)	
PFO closure yes or no	No (*n =* 125)	106 (106/165, 64.2%)	19 (19/22, 86.4%)	0.038
	Yes (*n =* 62)	59 (59/165, 35.8%)	3 (3/22, 13.6%)	
Follow-up after ≥12 months				
Imaging after the event	No (*n =* 149)	138 (138/165, 83.6%)	10 (10/22, 45.5%)	<0.001
	Yes (*n =* 39)	27 (27/165, 16.4%)	12 (12/22, 54.5%)	

Nominal variables in percent. Metric and ordinal variables with normal distribution with mean and standard deviation. If no normal distribution, median and 25th and 75th quartile. Significance level = 0.05. HbA1c, Hemoglobin A1c; ECG, Electrocardiogram; PFO, patent foramen ovale; TIA, transient ischemic attack.

This consideration is especially relevant in patients aged ≥60 years, a population in which both AF prevalence is higher and the predictive performance of established scores is limited. The RoPE score assigns a maximum of six points to this age group, effectively excluding older patients from the high-probability category irrespective of other favorable characteristics, while the PASCAL classification lacks validation in this demographic. Although older individuals were underrepresented in major meta-analyses, such as that by Mojadidi et al. (10), demographic trends and pathophysiological considerations suggest that selected older patients may still benefit from PFO closure, and rigid age-based exclusion appears unjustified.

Emerging evidence, including recent real-world data from Satpathy et al., supports the benefit of PFO closure in patients aged > 60 years, demonstrating a reduction in recurrent stroke risk while maintaining a favourable safety profile (23). Our findings complement this evidence by proposing a practical tool for risk stratification in this underrepresented population. Although our subgroup of patients ≥60 years was limited (*n =* 78), we identified a statistically significant AS5F cut-off value of 3.8 for discriminating AF development. This threshold could serve as an additional decision criterion: older patients with AS5F scores below 3.8 and favourable PFO morphology may represent a subgroup with a particularly advantageous benefit–risk profiles for closure, while those with scores above this threshold might benefit more from intensified AF screening and anticoagulation if AF is detected.

Analysis of recurrent ischemic events provides further insight into the complexity of stroke prevention in PFO patients. While PFO closure was associated with significantly lower recurrence rates, the etiology of recurrent events remained undetermined in over 50% of cases according to TOAST classification. This high proportion of cryptogenic recurrences—even after comprehensive diagnostic workup—highlights several important considerations:

First, it underscores the multifactorial nature of stroke risk in many patients, where PFO may coexist with other subtle or intermittent mechanisms such as paroxysmal AF, covert atrial cardiomyopathy, or low-grade atherosclerosis not meeting traditional TOAST criteria. The increased cardiovascular risk factor burden observed in patients with recurrent events supports this hypothesis and aligns with findings from Sørensen et al. (24). Second, it emphasizes the limitation of current diagnostic approaches in capturing all relevant stroke mechanisms, particularly intermittent phenomena like paroxysmal AF despite extended monitoring. Third, it suggests that even when PFO is not the sole mechanism, its closure may still contribute to risk reduction in a subset of patients by eliminating one pathway for paradoxical embolism.

While acknowledging the single-center design and moderate sample size, several methodological aspects strengthen the clinical relevance of our findings. The extended median follow-up of 46.4 months exceeds that of several randomized controlled trials and provides meaningful insight into long-term outcomes beyond the immediate peri-procedural period. The real-world setting reflects contemporary clinical practice more accurately than highly selected trial populations, capturing the heterogeneity and complexity of routine decision-making. The inclusion of both retrospectively identified and prospectively enrolled patients (*n =* 142 and *n =* 46, respectively) allowed for comprehensive capture of relevant cases while maintaining rigorous follow-up standards. Importantly, our study addresses a critical knowledge gap regarding patients aged ≥60 years underrepresented in landmark PFO closure trials. While Mojadidi et al.’s meta-analysis of 3,440 patients from major trials demonstrated clear benefit of PFO closure, individuals aged ≥60 years comprised less than 1% of participants (10). Our cohort included 78 patients in this age group, with 14 developing AF, providing one of the larger real-world datasets examining this specific population. Although larger multicenter studies are needed for definitive conclusions, our findings offer preliminary evidence supporting individualized assessment rather than blanket age-based exclusions (Table 6).

**Table 6:** Association of RoPE score and AS5F score with atrial fibrillation (AF): multivariable logistic regression and age-stratified analysis.

Analysis	Variable	aOR/Mean	95% CI/SD	*p*-value
Binary logistic regression (Model 1)	RoPE score on admission (0–10)	0.652	0.463–0.918	0.014
	AS5F (%)	1.125	0.985–1.284	0.082
Binary logistic regression (Model 2)	RoPE score on admission (0–10)	0.799	0.559–1.141	0.217
	AS5F (%)	1.106	0.952–1.284	0.187
	Enlarged left atrium	3.275	0.755–14.212	0.113
Age-stratified AS5F analysis	Age <60, AF no (*n =* 91)	1.330	±1.073	0.056
	Age <60, AF yes (*n =* 7)	0.700	±0.346	
	Age ≥60, AF no (*n =* 64)	3.738	±2.710	<0.001
	Age ≥60, AF yes (*n =* 14)	6.836	±5.794	

Nagelkerke R^2^ for Model 2 = 0.168. Significance level = 0.05. AF = atrial fibrillation; aOR, adjusted odds ratio; CI, confidence interval; AS5F, Age, Stroke Severity (NIH Stroke Scale >5) to Find AF; RoPE, Risk of Paradoxical Embolism. Mean values and ±SD are reported in %.

Our findings warrant validation in larger, multicenter cohorts with prospective data collection and standardized AF detection protocols. Specific areas for future research may include: determination of age-specific AS5F cut-offs for optimal discrimination; prospective studies comparing outcomes between AS5F-guided versus conventional decision-making; investigation of whether intensive AF monitoring guided by elevated AS5F scores improves detection rates and enables targeted anticoagulation; and cost-effectiveness analyses of incorporating AS5F assessment into routine practice.

### Limitations

Important limitations merit acknowledgment. The single-center design limits generalizability, although our institution serves a large catchment area with diverse patient demographics. The inclusion of both retrospectively identified (*n =* 142) and prospectively enrolled patients (*n =* 46) introduces the risk of heterogeneous data quality and ascertainment bias. A descriptive comparison of key baseline parameters between the two subgroups suggested that prospectively enrolled patients more frequently underwent PFO closure and had PFO more often attributed as the cause of the index event, likely reflecting improvements in diagnostic workup and interdisciplinary decision-making over the course of the study period rather than a systematic bias. Nevertheless, residual confounding from differential documentation practices cannot be fully excluded.

The loss of 27 patients (12.5% of the original cohort of 215) due to withdrawal of consent or loss to follow-up represents a relevant limitation. A descriptive analysis of available parameters in excluded patients revealed that all 27 were identified retrospectively, showed a lower rate of PFO closure, and had less complete data overall. A residual attrition bias therefore cannot be excluded and should be considered when interpreting follow-up outcomes.

The inclusion of TIA patients warrants careful interpretation, as PFO closure is not a guideline-supported indication following TIA. Furthermore, since DWI-MRI was not consistently available, a tissue-based reclassification of TIA could not be systematically applied, and some patients may have had minor strokes misclassified as TIA. Findings in this subgroup should therefore be regarded as exploratory and hypothesis-generating rather than practice-defining.

The moderate sample size, particularly in subgroup analyses, reduces statistical power for detecting associations and increases confidence interval widths. The relatively low number of AF episodes (*n =* 23) and recurrent strokes (*n =* 22) constrains the precision of effect estimates. The AS5F cut-off value of 3.8 identified in patients aged ≥60 years was derived from a limited subgroup (*n =* 78) and should be considered hypothesis-generating, requiring prospective validation before clinical implementation.

Heterogeneity in AF detection methods—ranging from standard ECG to extended Holter monitoring—introduces variability in AF ascertainment, potentially misclassifying some patients. The inclusion period spanning 2015–2022 encompasses evolving practice patterns and emerging evidence, potentially introducing temporal heterogeneity in decision-making. Data collection and follow-up concluded in November 2023; while the median follow-up of 46.4 months exceeds that of several landmark randomized trials, events occurring beyond this date are not captured.

Intrinsic limitations of the RoPE score (maximum score of 6 for patients aged ≥60 years) and the PASCAL classification (validated primarily in patients aged <60 years) restrict their applicability in older patients — the very group for whom additional decision tools are most needed.

Finally, absence of post-procedural echocardiographic data and device-specific information limits mechanistic insights into periprocedural AF and residual shunt.

## Conclusion

In summary, this real-world cohort study suggests that PFO management in clinical practice adheres to guideline recommendations, with RoPE and PASCAL scores serving as primary decision criteria.

The study’s novel contribution lies in proposing the AS5F score as a complementary risk stratification tool that addresses competing stroke etiologies, particularly AF. By identifying patients at higher AF risk who may derive limited benefit from PFO closure, the AS5F score could enhance individualized treatment decisions.

This approach may be particularly relevant in patients aged ≥60 years, where conventional PFO scores show reduced discriminatory capacity and the prevalence of alternative stroke mechanisms increases. However, given the exploratory nature of the AS5F cut-off value of 3.8 identified in this limited subgroup, these findings should be considered hypothesis-generating and require prospective validation in larger, dedicated cohorts before clinical implementation.

The substantial proportion of recurrent events with undetermined etiology underscores the complexity of stroke prevention in PFO patients and the need for comprehensive evaluation addressing multiple potential mechanisms. While larger prospective studies are needed for validation, integrating AS5F assessment into decision algorithms represents a pragmatic, evidence-informed approach to optimizing patient selection for PFO closure across all age groups, particularly in the growing population of older adults for whom current scoring systems provide insufficient guidance.

## Data Availability

The original contributions presented in the study are included in the article/supplementary material, further inquiries can be directed to the corresponding author.

## Glossary

aOR: Adjusted Odds Ratio
AF: Atrial Fibrillation
ASA: Atrial Septal Aneurysm
AS5F: Age, Stroke Severity (NIH Stroke Scale > 5) to Find AF
CI: Confidence Interval
DWI: Diffusion weighted imaging
ECG: Electrocardiogram
ESO: European Stroke Organisation
ESUS: Embolic Stroke of Undetermined Source
HbA1c: Hemoglobin A1c
IQR: Interquartile Range
LS: Large-Shunt (> 20 bubbles in the left atrium on transoesophageal echocardiogram)
MRI: Magnetic Resonance Imaging
mRS: modified Rankin Scale
PASCAL: PFO-Associated Stroke Causal Likelihood
PFO: Patent Foramen Ovale
RoPE: Risk of Paradoxical Embolism
ROC: Receiver Operating Characteristics
TIA: Transient Ischemic Attack
TOAST: Trial of Org 10,172 in Acute Stroke Treatment
